# Premature Interments

**Published:** 1846-05

**Authors:** 


*Royal College of Surgeons.*—Mr. J. H. Green has been elected a member of the Court of Examiners of the Royal College of Surgeons, to fill up the vacancy occasioned by the resignation of Sir Benjamin Brodie.—*London Med. Gaz.*

